# Human rabies associated with domestic cat exposures in South Africa, 1983–2018

**DOI:** 10.4102/jsava.v91i0.2036

**Published:** 2020-07-06

**Authors:** Antoinette A. Grobbelaar, Lucille H. Blumberg, Veerle Dermaux-Msimang, Chantel A. le Roux, Naazneen Moolla, Janusz T. Paweska, Jacqueline Weyer

**Affiliations:** 1 Center for Emerging Zoonotic and Parasitic Diseases, National Institute for Communicable Diseases of the National Health Laboratory Service, Johannesburg, South Africa; 2 Department of Tropical Diseases, University of Pretoria, Pretoria, South Africa; 3 Department of Medical Virology, Center for Viral Zoonoses, University of Pretoria, Pretoria, South Africa

## Introduction

Rabies is a fatal encephalitic disease caused by lyssaviruses belonging to the family Rhabdoviridae. At the time of this report, a total of 16 species of lyssaviruses, which included the prototype rabies virus (RABV), and 2 related but unclassified bat lyssaviruses, Taiwan and Kothalati, had been recognised by the International Committee on Taxonomy of Viruses (ICTV 2019). Globally RABV, also referred to as ‘classic rabies’, circulates in natural transmission cycles involving domestic dogs and various wildlife species. In the Americas, RABV is found in certain insectivorous and haematophagous bat species (Banyard et al. 2013). The public health burden of rabies is, however, very closely related to the occurrence of the disease in domestic dogs; thus, human cases of rabies are mostly reported from areas where dog rabies is uncontrolled (Hampson et al. 2015). An annual estimation of 59 000 human deaths occur worldwide with 95% of rabies cases occurring in Africa and Asia (Hampson et al. 2015). In South Africa, RABV circulates both in domestic animals and wildlife cycles, involving the canid and mongoose variants of the virus (Nel, Thomson & Von Teichman 1993). The urban cycle involves domestic dogs reported from various locations in the country, but particularly from the KwaZulu-Natal, Eastern Cape, Limpopo and Mpumalanga provinces (Cohen et al. 2007; Zulu, Sabeta & Nel 2009). Sylvatic cycles of the canid variant RABV in bat-eared foxes and black-backed jackal (Zulu et al. 2009) and the mongoose variant RABV in certain species of mongoose occur in South Africa (Van Zyl, Markotter & Nel 2010). Apart from the reservoir species, canid and mongoose RABV infections are reported in an array of domestic and wildlife species in the country, with these animals primarily serving as dead-end hosts (Sabeta et al. 2018). Laboratory-confirmed human rabies cases in South Africa are predominantly dog-mediated, and seven cases of rabies linked to other domestic species and wildlife have been reported (Weyer et al. 2011).

Increased bio-surveillance, particularly in bats, has led to the identification of several novel lyssaviruses during the last decade (Banyard & Fooks 2017; Hayman et al. 2016). Rabies in humans caused by rabies-related lyssavirus infections is rarely reported (Johnson et al. 2010). In South Africa, the occurrence of Duvenhage virus (DUVV) and Lagos bat virus (LBV) has been reported, respectively, associated with certain insectivorous and frugivorous bats (Markotter et al. 2006; Paweska et al. 2006). Two human cases of rabies in South Africa have been associated with bat exposures and DUVV infection (Paweska et al. 2006). To date, no cases of LBV infection in humans have been reported. Apart from these bat-associated lyssaviruses, the Mokola virus (MOKV) has also been reported from South Africa. Although found in a variety of terrestrial mammals, historically this virus has not been associated with bats. The MOKV has been reported in rabies cases involving domestic dogs, shrews (*Crocidura* spp.) and rodents (*Lophuromys* sp.), but most frequently domestic cats. Two cases of human rabies because of MOKV infection have been described, but the soundness of these reports has been questioned (Sabeta et al. 2007).

Globally, the occurrence of rabies in domestic cats is less commonly reported than in their fellow domesticated species, the dog. This holds true for South Africa too with rabies in cats reported at a much lower frequency than in domestic dogs (Department of Agriculture, Forestry and Fisheries 2019). It is noteworthy that there is no evidence to indicate that domestic cats are involved as natural reservoirs of the disease in South Africa or elsewhere and serve only as dead-end hosts. Interestingly, various cases of bat-associated RABV and rabies-related lyssavirus infections from domestic cats have been reported (Dacheux et al. 2009; Harris et al. 2006; McQuiston et al. 2001). In South Africa, cats have been associated with infections of MOKV and LBV (Coertse et al. 2017; Markotter et al. 2006).

This study reports on the occurrence of human rabies associated with domestic cat exposures in South Africa over a 36-year period, 1983–2018. Deoxyribonucleic acid (DNA) sequencing analysis was performed to determine the lyssavirus species involved in these cases.

## Materials and methods

### Cases and samples

The National Institute for Communicable Diseases (NICD) of the National Health Laboratory Service (NHLS) is the reference laboratory for the investigation of human rabies cases in South Africa. Case histories for human rabies cases in South Africa, since 1983, were archived at the laboratory and metadata captured in a routinely maintained case database. Clinical material and virus isolates derived during diagnostic investigations are available at the laboratory.

### Data extraction and analysis

Rabies case definitions and laboratory tests used for confirmation of cases were previously described (Weyer et al. 2011). Only laboratory confirmed rabies cases were considered for this study. Data for the source of exposure, age, sex and geographical location of exposure of cases were extracted from the routinely maintained case database at the NICD. The cat-associated rabies cases in humans were mapped based on global positioning system (GPS) coordinates of place of exposure by using Arcmap 10.2.2 (Esri, Redlands, CA, United States [US]).

### Ribonucleic acid extraction, polymerase chain reaction and sequencing

According to the manufacturers’ instructions, viral ribonucleic acid (RNA) was extracted from the Trizol^®^ (Invitrogen, Waltham, US) lysed clinical samples or virus isolates derived from clinical samples by using the QIAamp® Viral RNA mini kit (Qiagen, Hilden, Germany). Amplicons generated by using a cocktail of oligonucleotides specific for rabies and rabies-related viruses, targeting a portion of the nucleocapsid gene, were subjected to Sanger dideoxy nucleotide sequencing (Heaton et al. 1997). Briefly, amplicons were purified by using the Wizard® SV Gel and PCR clean-up System (Promega, Madison, Wisconsin, US) and the sequence determined by using a BigDye V3.1 Terminator Cycle Sequencing Ready Reaction kit (Applied Biosystems, Warrington, Great Britain) and a 3500XL Genetic Analyzer (Applied Biosystems, Foster City, CA, US).

### Deoxyribonucleic acid sequence analysis and phylogenetic analysis

Nucleotide sequences were edited by using BioEdit version 7.0.5.3 (Hall 1999), and alignments were generated by using the ClustalX Multiple Alignment analysis software as implemented in Molecular Evolutionary Genetics Analysis (MEGA)7.0.20 (Kumar, Stecher & Tamura 2016). Phylogenetic analysis was performed by using a maximum likelihood method of MEGA version 7 based on the General Time Reversible model under 1000 bootstrap iterations.

### Ethical consideration

Ethics clearance for the study was obtained through the protocol entitled: Essential communicable disease surveillance and outbreak investigation activities of the NICD, approved by the University of the Witwatersrand Human Ethics Committee (reference number: M160667).

## Results

**FIGURE 1:**
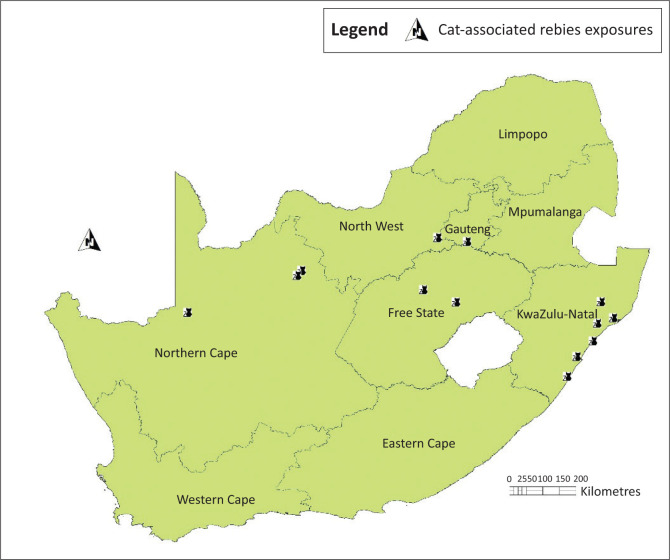
Location of confirmed human rabies cases associated with cat exposures in South Africa, 1983–2018.

From a total of 458 confirmed human rabies cases reported between 1983 and 2018 in South Africa, 13 (2.84%) cases were linked to domestic cat exposures. These cases involved both males (*n* = 7) and females (*n* = 6) with age ranging from 3 to 69 years. The cases were reported from KwaZulu-Natal (*n* = 6, 46.1%), Northern Cape (*n* = 3, 23.1%), Free State (*n* = 2, 15.4%), North West (*n* = 1, 7.7%) and Gauteng (*n* = 1, 7.7%) provinces (Figure 1).

**FIGURE 2:**
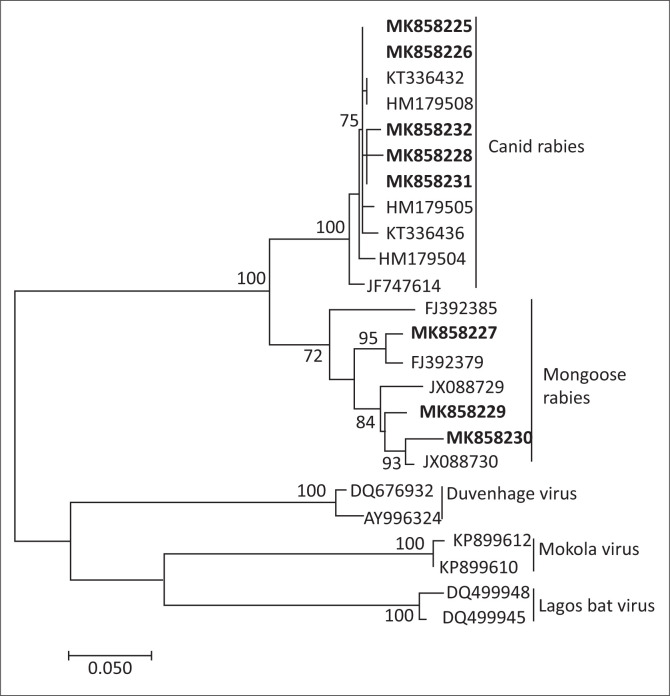
Maximum likelihood phylogenetic reconstruction of partial rabies nucleocapsid gene sequences from eight human rabies cases resulting from cat exposures in South Africa (indicated in boldface) and other Genbank sequences representing rabies-related and lyssavirus variants. Node values indicate bootstrap statistical support.

Of the 13 cases, eight clinical samples or virus isolates were available for further analysis in this study. Molecular sequencing of the partial nucleoprotein gene indicated that all eight cases were associated with RABV as summarised in Table 1. No cases of MOKV infection or other rabies-related viruses were identified. Phylogenetic analysis of the partial nucleoprotein sequence could be used to distinguish the variant of RABV involved (Figure 2). A total of five cases were typed as the canid variant, and the remaining three cases were resolved as the mongoose variant.

**TABLE 1:** Viruses included in phylogenetic analysis.

Genbank accession numbers	SA province	Year	Lyssavirus/variant
**MK858225**	**KwaZulu-Natal**	**1985**	**RABV/canid**
AY996324	Limpopo	1986	DUVV
**MK858226**	**KwaZulu-Natal**	**1988**	**RABV/canid**
FJ392385	Mpumalanga	1990	RABV/mongoose
FJ392379	Eastern Cape	1996	RABV/mongoose
**MK858227**	**Northern Cape**	**2002**	**RABV/mongoose**
HM179505	Mpumalanga	2004	RABV/canid
DQ499948	KwaZulu-Natal	2004	LBV
DQ499945	KwaZulu-Natal	2004	LBV
HM179508	Eastern Cape	2005	RABV/canid
HM179504	Limpopo	2006	RABV/canid
DQ676932	North-West	2006	DUVV
JX088729	Free state	2007	RABV/mongoose
JX088730	North-West	2007	RABV/mongoose
**MK858228**	**KwaZulu-Natal**	**2007**	**RABV/canid**
JF747614	KwaZulu-Natal	2008	RABV/canid
KT336432	North-West	2012	RABV/canid
KT336436	Gauteng	2012	RABV/canid
KP899610	KwaZulu-Natal	2012	MOKV
KP899612	KwaZulu-Natal	2014	MOKV
**MK858231**	**KwaZulu-Natal**	**2016**	**RABV/canid**
**MK858229**	**KwaZulu-Natal**	**2018**	**RABV/mongoose**
**MK858230**	**Free state**	**2018**	**RABV/mongoose**
**MK858232**	**Free State**	**2018**	**RABV/canid**

Note: Viruses analysed in this study are indicated in bold.

SA, South Africa; RABV, rabies virus; DUVV, Duvenhage virus; LBV, Lagos bat virus; MOKV, Mokola virus.

## Discussion and conclusion

Human rabies associated with domestic cat exposures in South Africa was a rare event during the study period. Despite close contact of the human population with domestic cats, less than 3% of human rabies cases in the 36-year period were associated with exposure to these animals.

Notably, it was found that rabies in domestic cats was linked to both canid and mongoose RABV cycles in the country. This study included only typing of RABVs associated with human rabies cases and hence does not necessarily offer an accurate estimation of the frequencies of the respective variants in cat rabies cases in the country. Systematic typing of RABVs associated with rabies in cats would offer an improved understanding of the frequency of canid and mongoose variant rabies in cats. Regardless, this is an important consideration for rabies control in the country particularly during the post-dog rabies elimination phase. The elimination of rabies in dogs, therefore, would not entirely remove the risk of rabies in cats, because the transmission of the virus from mongooses to cats has been demonstrated. Continued rabies vaccination of cats will remain important even after the elimination of rabies in domestic dogs has been achieved as spill over related to a sylvatic cycle of RABV in South Africa will continue to occur.
